# Comparison of pre-treatment with OCPs or estradiol valerate vs. no pre-treatment prior to GnRH antagonist used for IVF cycles: An RCT

**Published:** 2018-08

**Authors:** Ensieh Shahrokh Tehrani Nejad, Fatemeh Bakhtiari Ghaleh, Bita Eslami, Fedyeh Haghollahi, Maryam Bagheri, Masoumeh Masoumi

**Affiliations:** 1 *Vali-Asr Reproductive Health Research Center, Tehran University of Medical Sciences, Tehran, Iran*.; 2 *Breast Disease Research Center (BDRC), Tehran University of Medical Sciences, Tehran, Iran.*; 3 *Department of Reproductive Health, School of Nursing and Midwifery, Tehran University of Medical Sciences, Tehran, Iran.*

**Keywords:** IVF, GnRH antagonist, Oral contraceptives, Estradiol

## Abstract

**Background:**

Both oral contraceptive pills (OCPs) and estradiol valerate (E2) have been used to schedule a gonadotropin-releasing hormone antagonist in vitro fertilization (IVF) cycles. Since the suppression of follicle-stimulating hormone by OCPs can stay 5-7 days after stopping the pills, it seems that starting the gonadotropin-releasing hormone (GnRH) after 6 days of pre-treatment discontinuation may be important in IVF outcomes.

**Objective:**

The aim of the present study was to determine the number of mature oocyte and pregnancy rate of three pretreatment methods for fresh embryo transfer cycles.

**Materials and Methods:**

In this randomized controlled trial, two-hundred ten women (18-35 yr and less than 2 previous IVF attempts) undergoing IVF with the GnRH antagonist protocol were randomized to the OCP, E2, and no pretreatment arms. OCP group (n=53) received OCP (ethinyl estradiol30 μg and levonorgestrel150 μg), E2 group (n=63) received 4 mg/day oral E2 (17β‐E2) for 10 days from day 20 of the previous cycle and GnRH antagonist stimulation was started 6 days after the interruption of OCP and E2. The control group (n =70) did not receive any pretreatment.

**Results:**

No significant difference was observed in the mean number of the mature oocyte, endometrial thickness, and embryo quality. The pregnancy rate in E2 group was higher than the two other groups (42.9% vs 39.6% and 34.3% in OCP and control group, respectively), but the difference was not statistically significant (p=0.59).

**Conclusion:**

It seems OCP or E2 pretreatment could not improve the fresh IVF-embryo transfer outcomes.

## Introduction

Oral contraceptive pills (OCPs) and synthetic Estradiol has been used for many years to schedule ovarian stimulation which is started on a previous luteal cycle of IVF. This schedule makes it easy for stimulation and laboratory activities and prevents weekend oocyte retrieval (1-4). The results of the systematic review and meta-analysis in 2008 showed the probability of ongoing pregnancy was not significantly different between patients with and without OCP pretreatment. Although OCP pretreatment did not significantly alter the number of cumulus-oocyte complexes and fertilization rate (5), further studies are necessary for the more solid conclusion. Since the number of papers that investigated the different method of pretreatment is not enough and in most studies, the pill-free interval between the discontinuation of pills (OCP or estrogen) to start gonadotropin is 1-5 days (6). 

Since it acquired 5 days after stopping OCP for FSH to return to baseline levels from a strong suppression and this was an optimal success period in cycles pretreated with OCP (7). Also in the most studies, duration of pre-treatment usage is variable (4, 5, 8). So, it seems that the different time of administration protocol may be important. Therefore, in the current study, we evaluated the use of E2 and OCP pre-treatment with fixed 6 days interval to start the GnRH antagonist protocol. 

We conducted a prospective, randomized and pretreatment controlled trial in three groups of patients before GnRH antagonist protocol and comparing the IVF/ Intracytoplasmic sperm injection (ICSI) outcomes. 

## Materials and methods

Two hundred twenty-five women who attending the infertility center of Vali Asr hospital, and were candidate for IVF were included, Inclusion criteria included of Age 18-35 yr, body mass index (BMI) between 19 and 30 kg/m^2^, less than 2 previous IVF attempt, and anti-mullerian hormone (AMH) 1-6 ng/mL and fresh embryo transfer. Exclusion criteria were FSH more than 10 IU/I, antral follicle count less than 4, the existence of hydrosalpinx in ultrasonograghy, uterus disorders such as uterus fibroid, endocrine and ovarian disorder and polycystic ovarian syndrome.

**Figure 1:**
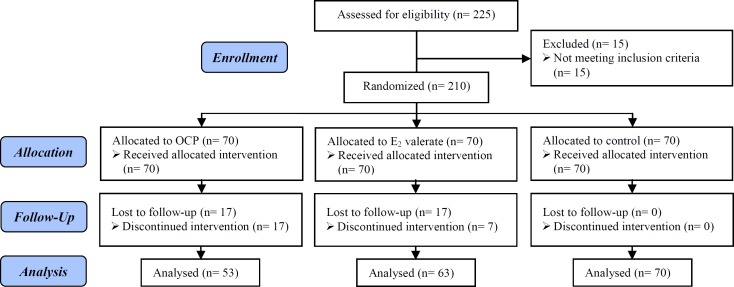
Consort Flowchart

Consort flowchart (Figure 1) shows 70 women allocated in each group. However, we had lost to follow up in both cases group. Therefore, we conducted analyses in 53, 63 and 70 patients in OCP, E2 valerate and control group, respectively.

All women were randomly allocated into three groups by sequentially numbered. Authors involved in data collection and data analysis were blinded to group assignment. The OCP group participants (n=53) started, the pill (30 μg of ethinyl E2 plus 150 μg of levonorgestrel [Maroline; Bayer Schering Pharma, Berlin, Germany]) for 10 days from the day 20 of the previous cycle, and stimulation with recombinant FSH was started 6 days after interruption of OCP. In patients allocated to the E2 group (n=63), pretreatment with E2 valerate tablet (Progynova; Schering, Berlin, Germany) was started from the day 20 of the previous cycle daily a dose of 4 mg (2 mg twice a day) orally for 10 days, and stimulation with recombinant FSH was started 6 days after interruption of E2 valerate. 

It should be noted that the fix 6 days after interruption of pretreatments was considered in two groups for the start of stimulation. If menstrual bleeding occurs in these duration time (1-6 days after interruption of E2 valerate or OCP), a daily dose of recombinant FSH (Gonal F; Merck Serono, Madrid, Spain) 150 IU (2 Vials, 75 Iu) subcutaneous was administrated in the three groups of study. In the absence of menstrual bleeding in this time (1-6 days after the interruption of E2 or OCP), the patient was excluded from the study. The control group (n=70) did not receive any pretreatment medication. Gonadotropin (Gonal F; Merck Serono, Madrid, Spain) was administrated in the second day of the natural cycle. In three groups, the GnRH antagonist (Cetrotide; EMD Serono, Switzerland) was introduced at a daily dose of 0.25 mg subcutaneously when the leading follicle reached 13 mm mean of diameter. 

Ovarian triggering was performed with 500 μg of recombinant human chorionic gonadotropin (hCG) (Ovitrelle; Merck Serono), which was administered as soon as two leading follicles reached more than or the mean diameter was equal to 17 mm. Ovum pickup was performed 36 hr later. ICSI was used to fertilize oocytes. A maximum of 2 embryos with top quality (A, B or AB) were transferred on day 3 by the catheter (Cook Medical, Ireland LTD) under the sterile condition (9). Luteal phase was supported with the micronized vaginal progesterone (Cyclogest 400; COX Pharmaceuticals, Bamstaple, UK) daily for 15 days, after the ovum pickup. All patients were monitored for ovarian follicular development and endometrial thickness by transvaginal ultrasound on the day of ovum pick up. The retrieved follicles in both ovaries and the endometrium thickness were recorded. The primary outcomes were the number of mature oocyte metaphase II, chemical and clinical pregnancy.

Clinical pregnancy was indorsed by transvaginal ultrasound 2 wk after positive beta-human chorionic gonadotropin. When an embryo does not grow after 12 Wk, it involves spontaneous abortion. Basal and laboratory information of patients was recorded by the chief investigator including age (yr), gravidity, BMI (kg/m^2^), infertility diagnosis, FSH level (mIU/mL), AMH level (ng/mL), and a number of previous IVF attempts. COS (Control of ovarian stimulation) parameters included the total days of GnRH-ant administration, peak endometrial thickness (mm), a total number of mature oocytes retrieved, and pregnancy rate (%). The number of cycles canceled was also noted. The total number of mature oocytes (metaphase II) and embryos (cleavage stage) were reported by the unique specialized embryologist of this center after pick up.

**Ethical consideration**

Informed consent was obtained from all participants included in the study. This paper was approved by Tehran University of medical sciences-IRAN (IR.TUMS.IKHC.REC.1395. 1964).

**Statistical analysis**

Continuous variables were compared with ANOVA-test and Kruskal-Wallis test between three groups by considering the normality of variables. Normality of variables was checked by Kolmogrov-Smirnov test (p>0.05). p<0.05 was considered to be statistically significant. If the differences will statistically significant, we will run post hoc tests. All statistical analysis was performed with the SPSS 20 package (SPSS, Inc., Chicago, IL, USA). By considering our protocol for sample size calculation, we estimated the incidence of pregnancy in intervention and control group will be 55% and 30%. Therefore, we calculated that 62 patients would be required in each group to detect a difference in outcome with a power 80% and α = 0.05 by using the Epi Info Web site (www.cdc.gov/epiinfo). 

## Results

The results manifested all groups in this study were comparable in terms of age, BMI, hormonal level, type, and cause of infertility (Table I). No significant differences were observed in the mean number of retrieved or matured oocyte, quality of embryo and chemical and clinical pregnancy (Table II). Although the pregnancy rate in E2 valerate group was higher than the other group (42.9% vs. 39.6% and 34.3% in OCP and control group), the differences were not statistically significant (p=0.59). Cycle cancellation, Spontaneous abortion, ovarian hyperstimulation syndrome and ectopic pregnancy were not seen in each group. We didn’t run post Test, because our results were not significantly difference.

**Table I:** Demographic characteristics of patients

**Characteristics**		**OCP (n= 53)**	**E**_2 _ **(n= 63)**	**Control (n= 70)**	**p-value**
Age (yrs)*		31.83 ± 3.65	31 ± 3.41	30.89 ± 4.09	0.34
BMI (kg/m2)*		24.02 ± 2.46	24.03 ± 2.38	24.11 ± 2.41	0.97
Duration of infertility (yrs)*		5.85 ± 2.99	6.64 ± 3.11	6.19 ± 3.53	0.24
AMH (ng/ml )*		3.05 ± 1.38	3.33 ± 1.80	3.66 ± 2.17	0.74
LH (IU/L )*		4.99 ± 2.54	4.77 ± 1.94	5.02 ± 2.50	0.33
FSH ( IU/L ) *		5.35 ± 2.11	5.34 ± 1.90	5.50 ± 2.026	0.40
Type of infertility**					
	Primary	40 (75.5)	46 (73)	59 (78)	0.26
	Secondary	13 (24.5)	17 (27)	11 (22)	
Cause of infertility**					
	Female	11 (20.8)	13 (20.6)	23 (32.9)	0.67
	Male	23 (43.4)	26 (41.3)	27 (38.6)	
	Both	6 (11.3)	9 (14.3)	6 (8.6)	
	Unknown	13 (24.5)	15 (23.8)	14 (20)	

ANOVA-test (Age, BMI, LH, FSH), Kruskal-wallis test (Duration of infertility, AMH), Chi-square test (Cause of infertility, Type of infertility)

OCP: Oral contraceptive pill

E2: Estradiol valerate

BMI: Body mass index

AMH: Anti-mullerian hormone

LH: Luteinize hormone

FSH: Follicle stimulating hormone

* Data presented as Mean ± SD,

** Data presented as n (%)

**Table II:** Stimulation cycle parameters

**Characteristics**		**OCP (n = 53)**	**E**_2 _ **(n = 63)**	**Control (n= 76)**	**p** ***-*** **value**
Stimulation days (n)*		9.38 ± 0.99	9.21 ± 0.95	9.35 ± 1.13	0.58
Total mature follicle (n)*		12.36 ± 3.77	12.56 ± 4.22	12.93 ± 4.77	0.71
Endometrial thickness (mm)*		9.51 ± 1.42	9.75 ± 1.51	9.73 ± 1.65	0.80
Retrieved mature oocytes (n)*		10.55 ± 3.38	10.71 ± 3.73	10.40 ± 4.38	0.92
The resulting embryos (n)*		7.94 ± 2.94	8.38 ± 3.26	8.06 ± 3.75	0.76
Quality of embryos (n)*					
	A	4.04 ± 1.72	4.22 ± 1.73	4.07±2.09	0.54
	AB	2 ± 0.82	2.21 ± 0.99	1.84 ± 1.06	0.16
	B	0.74 ± 0.71	0.67 ± 0.78	0.76 ± 0.75	0.67
	C	1.21 ± 0.77	1.29 ± 1	1.33 ± 0.83	0.66
Chemical pregnancy **		21 (39.6)	27 (42.9)	24 (34.3)	0.59
Clinical pregnancy **		21 (39.6)	27 (42.9)	24 (34.3)	0.59

Kruskal-wallis test (Stimulation days, total mature follicle, Endometrial thickness, Retrieved mature oocytes, Quality of embryos), Chi-squared (Chemical pregnancy, Clinical pregnancy)

OCP: Oral contraceptive pill

E2: Estradiol valerate

* Data presented as Mean ± SD,

** Data presented as n (%)

## Discussion

The results of the present study failed to show the statistically significant differences in pregnancy rate in IVF patients who received cycle scheduling with OCP, E2 valerate with a comparison to control group in a randomized clinical trial after 6 days of pretreatment discontinuation in GnRH antagonist cycles. The OCPs impact on IVF-ET cycles has been well studied in normal responders (5, 10-13), poor responders (14, 15) and hyper-responders (16). 

In some studies, the effect of OCP pretreatment on the results of IVF-ET has been reported in normal responders. The lower oocyte retrieval, clinical pregnancy, and live birth rates were reported (5, 7, 13) compared with the patients who had not received any pretreatment, while in the other studies have not shown these findings (10-16). Changes in results may be attributed to the use of different OCPs with a difference in duration of use (7, 17). However, the large majority of studies emphasize that OCP pretreatment was associated with a longer duration of Controlled ovarian stimulation (COS) and higher levels of gonadotropin utilization (6, 12, 13).

The results of the present study are consistent with the previously published Hauzman (7), Cedrin-Durnerin (18), and Griesinger (5, 13) findings that did not show differences in stimulation results between E2 and OCP groups. At the same time, our findings showed that there was no significant difference in the duration of COS and the use of gonadotropins in the E2, OCP and the third group (no pretreatment). The Hauzman study in the year of 2013 compared two groups of pretreatment with OCP and E2 valerate in IVF cycles. Their results showed no statistically significant differences in pregnancy rates (7). 

Another study by Cedrin-Durnerin showed 17-β estradiol or no pretreatment before daily recombinant FSH administration started on the first day of estrogen discontinuation or on cycle day 2 in non-pretreated women does not affect pregnancy rates, the number of retrieved oocyte and cycle outcomes (18).

According to the researcher's opinion, a 1-day washout period was too short to permit completely recovery of standard FSH levels (7). This led to an increase in gonadotropin consumption compared to cycles without any pretreatment. However, no clinical studies have been performed with COS started 6 days after stopping E2 administration. Also in the most studies, the starting and duration of pre-treatment day are variable (4, 5, 8).

The inconsistent results may be due to the difference in sample sizes, duration of pre-treatment utilization, and the E2 and OCP products utilized in the other studies. Barad showed that prior to COS, patients were used OCPs, with higher androgenic properties, have been shown to have lower oocyte retrieval than those using anti-androgenic OCPs or those not using OCPs (19). Currently, the present study contains information that OCP or E2 may not be required prior to the treatment of patients with IVF candidates because they do not increase the overall pregnancy outcomes of fresh IVF-ET cycles.

This study addresses a relevant answer to the question of how to manage ovulatory suppression in an IVF protocol. The advantages of this study were, 1-It compares three groups, included two treatment groups and one group without pretreatment as a control group. 2-To the best of our knowledge, this study is the first study to directly compare these two methods of cycle scheduling after the 6 days of discontinuation, and the fixed 10 days duration of pretreatment usage before GnRH antagonist cycles. Therefore, in this study, with the uniformity of these days, we eliminated this confounding factor to eliminate the possible side effects of drugs on the endometrium.

The main limitation of the present study is the considerable loss to follow up in both treated groups and we conducted treated analysis, the sample size and statistical power of our study would be decreased and these hypotheses need prospective validation in studies with adequate sample size. Ongoing pregnancy was not evaluated in this study and this was another limitation.

## Conclusion

Moreover, the results of this study showed that no significant differences were observed in the mean number of the matured oocyte, quality of embryo; chemical and clinical pregnancy. Therefore, OCP or E2 pretreatment only can be used for scheduling reasons and couldn’t improve the fresh IVF-ET outcomes.

## Note

***This article extracted from M.D. Thesis. (Fatemeh Bakhtiari Ghaleh)***

***Registration ID in RCT: IRCT2017020520351N9***
